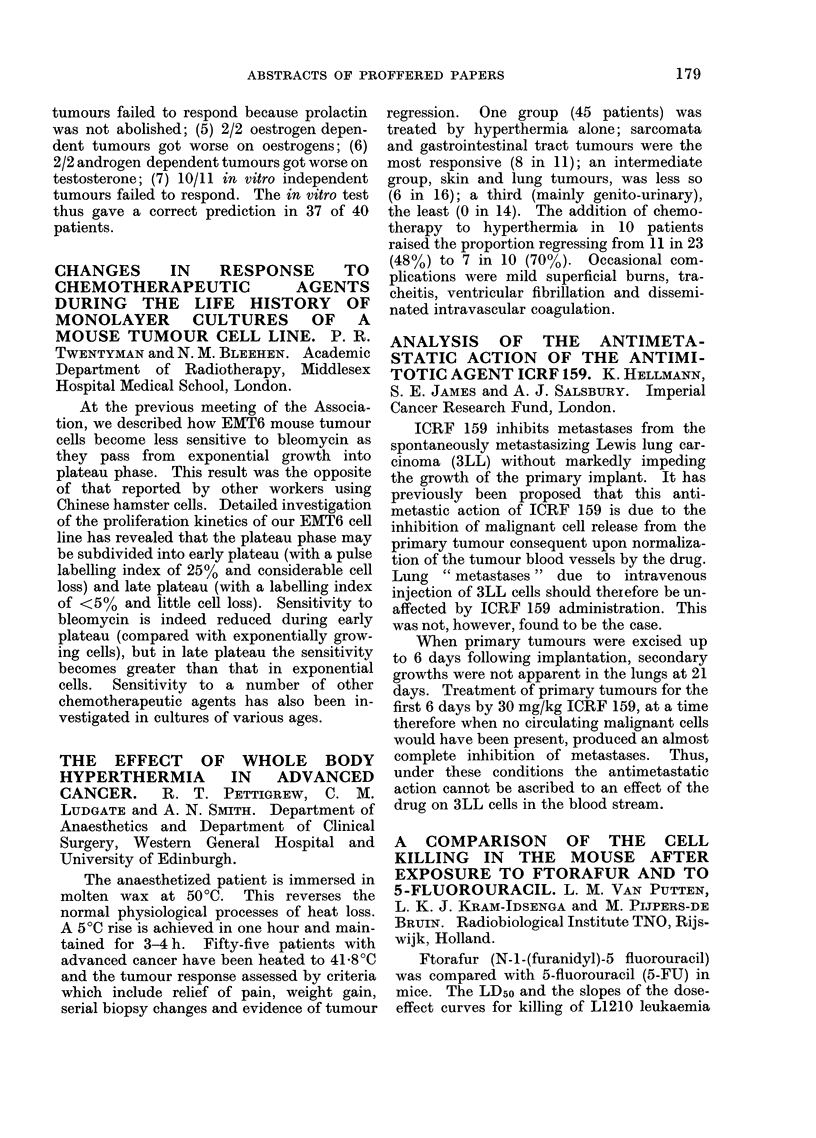# Proceedings: The effect of whole body hyperthermia in advanced cancer.

**DOI:** 10.1038/bjc.1974.152

**Published:** 1974-08

**Authors:** R. T. Pettigrew, C. M. Ludgate, A. N. Smith


**THE EFFECT OF WHOLE BODY HYPERTHERMIA IN ADVANCED CANCER.** R. T. Pettigrew, C. M. Ludgate and A. N. Smith. Department of Anaesthetics and Department of Clinical Surgery, Western General Hospital and University of Edinburgh.

The anaesthetized patient is immersed in molten wax at 50°C. This reverses the normal physiological processes of heat loss. A 5°C rise is achieved in one hour and maintained for 3–4 h. Fifty-five patients with advanced cancer have been heated to 41·8°C and the tumour response assessed by criteria which include relief of pain, weight gain, serial biopsy changes and evidence of tumour regression. One group (45 patients) was treated by hyperthermia alone; sarcomata and gastrointestinal tract tumours were the most responsive (8 in 11); an intermediate group, skin and lung tumours, was less so (6 in 16); a third (mainly genito-urinary), the least (0 in 14). The addition of chemotherapy to hyperthermia in 10 patients raised the proportion regressing from 11 in 23 (48%) to 7 in 10 (70%). Occasional complications were mild superficial burns, tracheitis, ventricular fibrillation and disseminated intravascular coagulation.